# Effect of a Concurrent Training Program with and Without Metformin Treatment on Metabolic Markers and Cardiorespiratory Fitness in Individuals with Insulin Resistance: A Retrospective Analysis

**DOI:** 10.3390/biom14111470

**Published:** 2024-11-19

**Authors:** Jairo Azócar-Gallardo, Alex Ojeda-Aravena, Eduardo Báez-San Martín, Tomás Herrera-Valenzuela, Marcelo Tuesta, Luis González-Rojas, Bibiana Calvo-Rico, José Manuel García-García

**Affiliations:** 1 Facultad de Ciencias del Deporte, Universidad de Castilla-La Mancha (UCLM), 45071 Toledo, Spain; bibiana.calvo@uclm.es (B.C.-R.); josemanuel.garcia@uclm.es (J.M.G.-G.); 2 Programa de Investigación en Deporte, Sociedad y Buen Vivir (DSBv), Universidad de Los Lagos, Osorno 5290000, Chile; 3 Departamento de Ciencias de la Actividad Física, Universidad de Los Lagos, Osorno 5290000, Chile; 4 Dirección de Docencia, Universidad de Los Lagos, Osorno 5290000, Chile; 5 Carrera de Entrenador Deportivo, Escuela de Educación, Universidad Viña del Mar, Viña del Mar 2580022, Chile; eduardo.baez@upla.cl; 6 Laboratorio de Evaluación y Prescripción de Ejercicio, Facultad de Ciencias de la Actividad Física y del Deporte, Universidad de Playa Ancha, Valparaíso 2340000, Chile; 7 School of Physical Activity, Sports and Health Sciences, Faculty of Medical Sciences, Universidad de Santiago, Santiago 7591538, Chile; tomas.herrera@usach.cl; 8 Exercise and Rehabilitation Sciences Institute, School of Physical Therapy, Faculty of Rehabilitation Sciences, Universidad Andres Bello, Santiago 7591538, Chile; marcelo.tuesta@unab.cl; 9 Laboratory of Sports Sciences, Sports Medicine Centre Sports MD, Viña del Mar 2580022, Chile; 10 Centro Tratamiento de la Obesidad, Pontificia Universidad Católica de Chile, Santiago 8320165, Chile; rojas.gonzalez.luis@gmail.com

**Keywords:** physical and rehabilitation medicine, pharmacologic treatment, cardiorespiratory fitness, insulin sensitivity, retrospective studies

## Abstract

Background: Type 2 diabetes mellitus is a metabolic disorder characterized by insulin resistance (IR), which is prevalent worldwide and has significant adverse health effects. Metformin is commonly prescribed as a pharmacological treatment. Physical exercise is also recognized as an effective regulator of glycemia, independent of metformin. However, the effects of inter-day concurrent training (CT)—which includes both endurance and resistance exercises—combined with metformin treatment on metabolic markers and cardiorespiratory fitness in individuals with IR remain controversial. Objective: This study aimed to analyze the effects of a 12-week inter-day CT program on metabolic markers and cardiorespiratory fitness in overweight/obese individuals with IR, both with and without metformin treatment. Additionally, inter-individual responses to CT were examined. Materials and Methods: Data from the 2022–2023 Obesity Center database were retrospectively analyzed. According to the eligibility criteria, 20 overweight/obese individuals diagnosed with IR participated in a 12-week CT program (three weekly sessions: two endurance and one resistance exercise session). Participants were divided into three groups: the exercise group (E-G: n = 7, 32.86 ± 8.32 years, 85.2 ± 19.67 kg), the exercise–metformin group (E-MG: n = 6, 34.83 ± 12.91 years, 88.13 ± 12.66 kg), and the metformin-only control group (M-G: n = 7, 34.43 ± 13.96 years, 94.23 ± 13.93 kg). The M-G did not perform physical exercise during the 12 weeks but continued pharmacological treatment. Body composition, metabolic markers, and cardiorespiratory fitness were assessed before and after the 12-week CT program. Results: A group-by-time interaction was observed for fasting insulin (F_2,17_ = 34.059, *p* < 0.001, η^2^_p_ = 0.88), the Homeostatic Model Assessment of Insulin Resistance (HOMA-IR) (F_2,17_ = 35.597, *p* < 0.001, η^2^_p_ = 0.80), and maximal fat oxidation (MFO) (F_2,17_ = 4.541, *p* = 0.026, η^2^_p_ = 0.348) following the CT program. The maximal oxygen uptake (VO2_max_) showed significant improvements in the E-G (F = 4.888, *p* = 0.041, ∆+13.3%). Additionally, the percentage of fat mass (%FM) and body mass (BM) were significantly reduced across all groups (F = 125.244, *p* < 0.001 and F = 91.130, *p* < 0.001, respectively). The BM decreased by ∆−9.43% in the E-G (five responders, Rs), ∆+9.21% in the EM-G (5 Rs), and ∆+5.15% in the M-G (3 Rs). The %FM was reduced in the E-G by ∆−22.52% (seven Rs). Fasting insulin and the HOMA-IR significantly improved in both the E-G and EM-G, with fasting insulin showing a ∆−82.1% reduction in the E-G (five Rs) and a ∆−85% reduction in the EM-G (six Rs). Similarly, the HOMA-IR improved by ∆+82.6% in the E-G (three Rs) and by ∆+84.6% in the EM-G (six Rs). Conclusions: The 12-week inter-day concurrent training program, whether combined with metformin or not, was similarly effective in improving metabolic markers in patients with insulin resistance as metformin treatment alone. Both exercise groups demonstrated a significant reduction in insulin sensitivity and an increase in maximal fat oxidation. Meanwhile, exclusive pharmacological treatment with metformin markedly decreased cardiorespiratory fitness, and consequently, fat oxidation.

## 1. Introduction

Obesity and hyperlipidemia exert a negative impact on insulin sensitivity, significantly impairing glucose transport through insulin-dependent tissues such as skeletal muscle, adipose tissue, and the liver [1,2]. As a compensatory mechanism, the pancreas increases insulin secretion into the bloodstream, leading to hyperinsulinemia and/or insulin resistance (IR) [3]. IR subsequently results in pancreatic β-cell dysfunction and impaired glycemic control, which promote the development of Type 2 Diabetes [3,4].

A key predictive factor for altered insulin sensitivity is the muscle’s ability to oxidize fatty acids [5]. Additionally, individuals with IR are expected to exhibit a decreased mitochondrial number and function, as well as a reduced muscle oxidative capacity [6]. As a result, cardiorespiratory fitness is expected to be compromised, further promoting sedentary behavior, weight gain, and hyperlipidemia.

Metformin and physical exercise are recognized as first-line treatments for IR [7] and Type 2 Diabetes [8]. Both interventions enhance glucose uptake in insulin-dependent tissues, thereby improving insulin sensitivity [9,10,11]. Endurance exercise increases cardiorespiratory fitness and mitochondrial function (e.g., increased fat oxidation), while decreasing circulating lipids (e.g., triacylglycerol and low-density lipoprotein) and blood pressure [1]. The chronic effects of endurance or aerobic- and resistance-exercise-based programs include increased concentrations of the glucose transporter protein receptor (GLUT–4) at the plasma membrane and sarcoplasmic reticulum, thereby enhancing the treatment of IR by improving glucose uptake in skeletal muscle cells [12,13,14,15].

Despite the benefits of metformin and physical exercise, the interaction between these treatments yields controversial results [16,17,18,19]. Malin and Braun [20] suggested that metformin attenuates exercise-induced improvements in glucose homeostasis, muscle mass, strength production [21], and cardiorespiratory fitness [22,23]. However, Ortega et al. [24] and Boulé et al. [18] reported no detrimental chronic effects of physical exercise on insulin sensitivity improvement in metformin-treated IR patients.

Endurance and resistance exercises are recommended by clinical guidelines for diabetes (e.g., American Diabetes Association and American College of Sports Medicine) and sedentary lifestyles (e.g., World Health Organization) [25]. Nevertheless, the impact of inter-day concurrent training (CT) with or without metformin treatment on insulin sensitivity in individuals remains unclear [25,26]. Malin and Braun [27] demonstrated that sedentary adults who participated in CT for 10 weeks without metformin (placebo) exhibited higher insulin sensitivity than those treated with metformin. However, no significant increases in maximal oxygen uptake (VO2_max_) or fat oxidation rates were observed.

Current evidence underscores the need to analyze the effects of CT in conjunction with metformin treatment in individuals with IR and to assess the inter-individual variability in CT responses. Álvarez et al. [28] investigated the effects and prevalence of individual responses to high-intensity interval and resistance training over 12 weeks in women with overweight/obesity and IR. Following the intervention, significant reductions in fasting glucose, insulin, and HOMA-IR levels were observed in both the high-intensity interval training and resistance training groups. However, no differences were found in the prevalence of non-responders between high-intensity interval training and lower-body resistance training regarding fasting glucose levels.

Therefore, this study aimed to analyze the effect of a 12-week inter-day CT program on metabolic markers and cardiorespiratory fitness in overweight/obese individuals with IR, treated with and without metformin. Additionally, inter-individual responses to CT were examined. We hypothesized that the combination of CT without metformin would have a more significant impact on metabolic markers and cardiorespiratory fitness in overweight or obese individuals with IR than CT combined with metformin [18,20,21,22,24,29].

## 2. Material and Methods

### 2.1. Design and Participants

This retrospective study analyzed data from 20 individuals (15 women and 5 men) from an obesity treatment center in Chile. Participants were included if they met the following criteria: (a) diagnosis of overweight or obesity with a body mass index (BMI) ≥ 25 kg/m^2^, (b) confirmed insulin resistance (HOMA-IR: 2.5–5.0) [30], and (c) physical inactivity as per the International Physical Activity Questionnaire [31]. Exclusion criteria comprised use of medications other than metformin, smoking, and incomplete assessments within the designated timeframe.

Participants were allocated into three groups: the exercise group (E-G: n = 7, age = 32.86 ± 8.32 years, height = 165.14 ± 10.53 cm, body mass = 85.2 ± 19.67 kg), exercise-metformin group (EM-G: n = 6, age = 34.83 ± 12.91 years, height = 159.00 ± 7.87 cm, body mass = 88.13 ± 12.66 kg), and metformin-only control group (M-G: n = 7, age = 34.43 ± 13.96 years, height = 165.76 ± 7.24 cm, body mass = 94.23 ± 13.93 kg).

To ensure study validity, the attending physician and physiotherapist conducted a comprehensive review of the center’s database from January 2022 to December 2023. Eligibility was determined based on voluntary participation and full attendance at the 12-week CT sessions, or completion of baseline and final assessments only. Participants self-reported physical inactivity via telephone contact during the 12-week period. The main researcher, blinded to the data, verified and exported the selected data to an Excel spreadsheet for analysis.

The study adhered to the ethical standards outlined in the Declaration of Helsinki and STROBE guidelines for observational studies [32], as well as recommendations for retrospective health studies [33]. The local ethics committee approved the study protocol and data processing (registration number: 151007005).

### 2.2. Assessments

Assessments were conducted independently for each participant by the treating physician and physiotherapist following standardized procedures. Evaluations occurred after enrollment, one week before, and one week after the 12-week CT period. Day one comprised fasting glucose and insulin, and body composition measurements. Day two involved monitoring maximal fat oxidation (MFO) and VO2_max_, and heart rate during cardiorespiratory testing.

All assessments were performed under consistent environmental conditions (21–23 °C) between 08:00 and 10:00 to minimize circadian effects. Medical professionals assessed fasting glucose and insulin levels, while a physiotherapist evaluated VO2_max_, MFO, and body composition.

#### 2.2.1. Body Composition

Height was measured using a stadiometer (accuracy, 0.5 cm). Body mass (BM) and the percentage of fat mass (%FM) were assessed via multifrequency octopolar bioimpedance (InBody 720; Seoul, Korea) after 6 h of fasting. For female participants, measurements were taken post-menstruation and after at least 12 h without exercise.

#### 2.2.2. Insulin Sensitivity (IS)

Insulin sensitivity was evaluated using the Homeostatic Model Assessment of Insulin Resistance (HOMA-IR), calculated using the formula developed by Matthews et al. [34]: (fasting insulin × fasting glucose)/405. Participants with HOMA-IR scores between 2.5 and 5.0 were classified as insulin resistant, in line with previous research conducted in Chile [30].

#### 2.2.3. Cardiorespiratory Fitness and Maximal Fat Oxidation

Participants were familiarized with test procedures beforehand. On the day of assessment, they arrived at the laboratory after fasting for 6–12 h and abstaining from alcohol, coffee, drugs (including metformin), and other stimulants for 24 h. They then performed an incremental test on a bicycle ergometer (Technogym Bike Med, Technogym^®^, Cesena, Italy), adapted from previous recommendations [35].

The theoretical maximum load (W) was estimated using the Jones et al. equation [36]. The protocol consisted of a 3 min rest period, a 3 min warm-up at 20% of the maximum load, followed by 6 min stages at 30, 40, 50, and 60% of the maximum load until a respiratory exchange ratio <1 was achieved. Subsequent 6 min stages continued until maximal effort was reached, with verbal encouragement provided.

The test was deemed maximal if the respiratory exchange ratio was ≥1.1 and/or if the maximum heart rate (HRmax) met or exceeded the theoretical maximum predicted by the Morris equation [37] for the bicycle ergometer test. Based on the last completed stage, the following variables were calculated using the mean of the last 30 s of exhaled air via a breath-by-breath gas analysis (Metalyzer 3B-R2, Cortex^®^, Leipzig, Germany): ventilatory threshold 2 (provided by MetaSoft^®^ Studio software version 5.9 and validated by visual inspection), HR_max_ (beats per minute), and maximum load (watts). The VO2_max_ (L/min) was determined using the same methodology. The MFO rate (g/h) was measured during exercise using the equations of Frayn (mean value of the O_2_ and CO_2_ volumes of the last 2 min of each completed 6 min stage) [38].

### 2.3. Inter-Day Concurrent Training Program

The 12-week inter-day CT program consisted of three distinct weekly sessions meticulously programmed by the main researcher and physiotherapist [39,40]. Sessions 1 and 3 focused on endurance exercises, while session 2 emphasized resistance exercises. A 48-h recovery period was ensured between sessions. Each session lasted 60–75 min and was conducted under the supervision of a physiotherapist from the obesity treatment center. Sessions were personalized for each participant to ensure tailored benefits.

All physical exercise sessions began with a 15 min warm-up, comprising 5 min of cycloergometer exercise at 65% of the VO2_max_, followed by joint mobility exercises and dynamic stretching. This structured warm-up aimed at preparing participants for the ensuing exercise types and maximizing safety and effectiveness.

Endurance exercise sessions were based on continuous cycloergometer exercise, with intensities ranging from 65–85% of the VO2_max_, monitored using heart rate telemetry (Polar T31, Polar, Kempele, Finland). The endurance exercise intensity was prescribed based on the training heart rate derived from a cardiorespiratory test to obtain the VO2_max_. This allowed for progressive overload throughout the 12 weeks by adjusting to the individual’s adaptive response to the training stimulus.

The program progression was as follows:

- Weeks 1–3: 50 min of continuous running at 65% VO2_max_.
- Weeks 4–6: third session changed to 20 min interval-based approach (1 min intervals at 85% VO2_max_; 3 min active recovery at 65% VO2_max_).
- Weeks 7–9: 20 min intervals with 2 min intervals at 85% and 2 min of active recovery at 65% VO2_max_.
- Weeks 10–12: return to original interval structure (1 min intervals; 1 min recovery).

The second weekly session focused exclusively on resistance training, targeting both upper body (chest press, latissimus pull-down, biceps curl) and lower body (leg press, prone femoral curl, leg extension) exercises. Participants performed 6 resistance exercises in each session, alternating between upper- and lower-body exercises with 30–60 s recovery periods. This circuit was repeated 3 times per session.

In the initial three weeks, the Rating of Perceived Exertion (RPE) scale (0–10) guided training intensity, with a target zone of 7–8. After 3, 6, and 9 weeks, the one-repetition maximum (1-RM) was measured as described by Jimenez and De Paz [41] and LeSuer et al. [42] to adjust resistance exercise loads and prescribe intensity as a percentage of 1-RM (target zone: 50–60%).

### 2.4. Statistical Analysis

Data are presented as mean and standard deviation. A Shapiro–Wilk normality test confirmed the normal distribution of all data. Levene’s test confirmed homoscedasticity. A repeated-measures ANOVA analyzed the effects of time (pre- and post-intervention) and group (E-G, E-MG, and M-G) factors. Post hoc pairwise comparisons (Bonferroni-adjusted) identified the source of significant differences. Effect sizes (ESs) were calculated using partial eta-squared (η^2^_p_) values. Cohen’s d was calculated as a measure of the effect size (ES), with threshold values as follows: <0.20 (trivial), 0.20–0.59 (small), 0.60–1.19 (moderate), 1.20–1.90 (large), 2.0–3.9 (very large), and > 4.0 (extremely large) [43]. The percentage delta (Δ%) expressed the pre- and post-intervention changes.

Participants’ inter-individual responses were classified as responders (Rs) and non-responders (NRs), defined as individuals demonstrating a change in physical fitness greater than twice the technical error (TE) away from zero [44]. TE was calculated using the equation established by Bonafiglia et al. [45]. Each metabolic assessment was performed pre- and post-intervention to calculate the TE. A change in the TE of more than twice represented a high probability (i.e., 12:1 odds ratio) that the observed response was a true physiological adaptation beyond what could have been expected owing to technical and/or biological variability.

The TE values were as follows: [body mass (kg), 2.458 × 2; fat mass percentage, 1.988 × 2 (%); fasting insulin, 5.701 × 2 (mg/dl); fasting glycemia, 2.871 × 2 (mg/dl; HOMA-IR, 1.414 × 2; VO2_max_, 0.181 (L/min) × 2; MFO, 4.164 × 2 (g/h)]. Fisher’s exact test was used for comparisons between groups [46]. All statistical analyses were performed using GraphPad PRISM (version 6.0, San Diego, CA, USA).

## 3. Results

### 3.1. Metabolic and Fitness Responses

The analysis revealed significant group-by-time interactions for fasting insulin (F_2,17_ = 34.059, *p* < 0.001, η^2^_p_ = 0.88), the HOMA-IR F_2,17_ = 35.597, *p* < 0.001, η^2^_p_ = 0.80), and MFO (F_2,17_ = 4.541, *p* = 0.026, η^2^_p_ = 0.348) following the CT program (Table 1).

Fasting insulin levels decreased significantly in both the E-G (−82.1%, ES = 4.06, very large) and the EM-G (−85%, ES = 4.54, very large), while no improvement was observed in the M-G. The HOMA-IR showed substantial reductions in the E-G (−82.6%, ES = 3.91, very large) and EM-G (−84.6%, ES = 3.69, very large) groups. The E-G demonstrated a significant increase in MFO of +181.1% (ES = 1.38, large).

BM exhibited significant time effects (F = 91.30, *p* < 0.001), with decreases observed across all groups: E-G (−9.43%), EM-G (−9.21%), and M-G (−5.15%). The ES was more pronounced in the E-G (ES = 0.73, moderate) compared to the EM-G (ES = 0.05, trivial) and M-G (ES = 0.30, small).

The %FM showed a significant time effect (F = 125.244; *p* < 0.001). The E-G demonstrated the most substantial improvement, with a reduction of −22.52% (ES = 0.83, large) compared to the M-G (−11.87%, ES = 0.45, moderate) and EM-G (−11.65%, ES = 0.63, moderate).

For the VO2_max_, a significant time effect was observed (F_2,17_ = 4.888, *p* = 0.041). The E-G improved cardiorespiratory fitness by +13.3% (ES = 0.30, moderate), while the EM-G showed a modest improvement of +5.26% (ES = 0.16, trivial). Notably, the M-G experienced a decrease of −30.8% (ES = 0.05, trivial).

**Figure 1:**
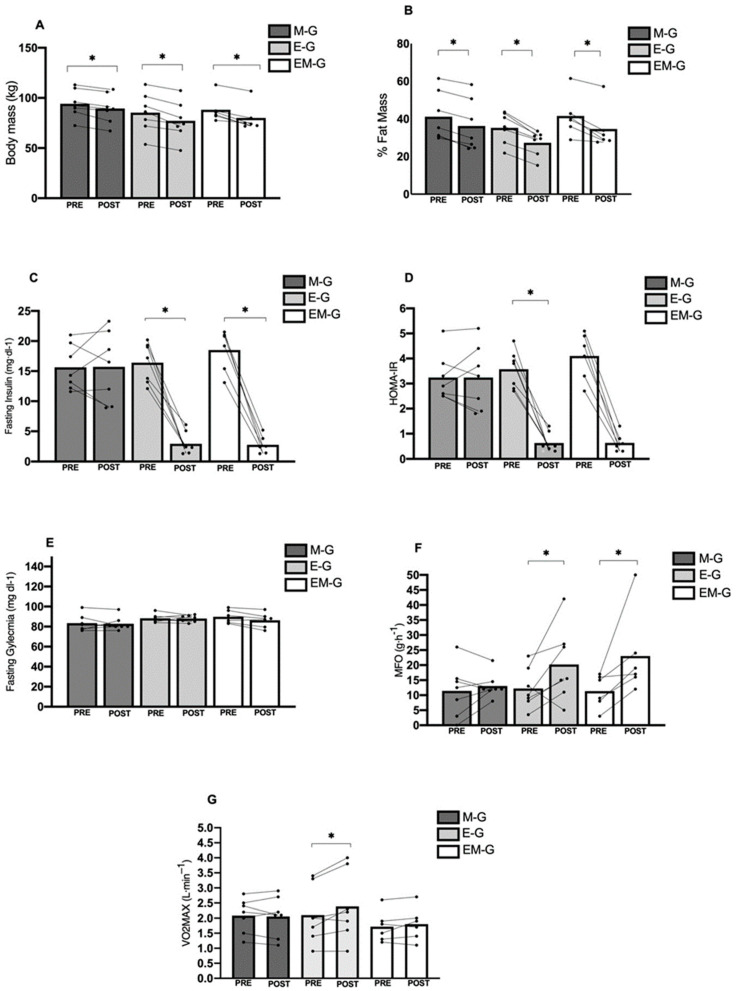
Group and individual changes before and after the concurrent training program for all group. *: represents statistically changes. (**A**) body mass; (**B**) %fat mass, (**C**) Fasting Insulin; (**D**) HOMA-IR; (**E**) Fasting Glycemia; (**F**) Maximal Fat Oxidation; (**G**) Maximal Oxygen Uptake.

Fasting blood glucose levels did not vary significantly between the study groups (F_2,17_ = 1.318, *p* = 0.294, η^2^_p_ = 0.134), although the E-G and EM-G demonstrated comparable improvements of +82.6% and +84.6%, respectively (ES = 3.91 and 3.69, respectively) (Figure 1).

### 3.2. Inter-Individual Responses

For BM, the M-G presented the lowest proportion of responders (Rs) at 33.3% (n = 3), compared to the E-G at 71.4% (n = 5) and the EM-G at 83.3% (n = 5). No significant differences were observed in the proportion of Rs between the groups. Regarding the %FM, the M-G had no Rs, whereas the E-G had 100% of the Rs (n = 7), and the EM-G had 66.7% of the Rs (n = 4).

Fasting insulin levels were not recorded in the M-G; however, the E-G had a 71.1% response rate (n = 5), and the EM-G had a 100% response rate (n = 5). Significant differences in R ratios were observed between the M-G and E-G (*p* = 0.004) and between the M-G and EM-G (*p* < 0.001). For the HOMA-IR, the M-G showed no Rs, whereas the E-G had 42.8% of the Rs (n = 3), and the EM-G had a 100% response rate (n = 3). A significant difference was observed between the Rs values of the M-G and EM-G.

**Figure 2:**
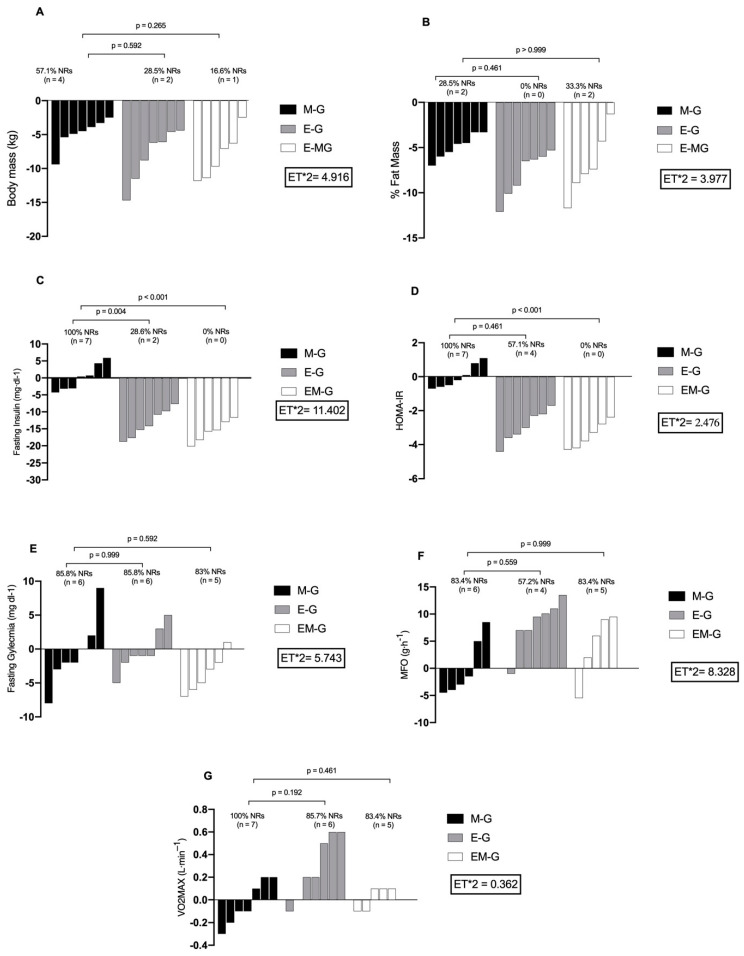
Inter-individual variability to concurrent training in the groups analyzed. (**A**) body mass; (**B**) %fat mass, (**C**) Fasting Insulin; (**D**) HOMA-IR; (**E**) Fasting Glycemia; (**F**) Maximal Fat Oxidation; (**G**) Maximal Oxygen Uptake.

For fasting glycemia, the M-G had 28.5% of the Rs (n = 2), whereas both the E-G and EM-G had 50% of the Rs (n = 3). For the VO2_max_, no Rs were recorded in the M-G, whereas the E-G showed 42.8% of the Rs (n = 3) and the EM-G showed 16.6% of the Rs (n = 1) (Figure 2).

## 4. Discussion

This study aimed to analyze the effects of a 12-week inter-day CT program on metabolic markers and cardiorespiratory fitness in overweight/obese individuals with IR, treated with or without metformin. Additionally, we examined inter-individual responses to CT. The main results revealed significant improvements in both the metformin and non-metformin training groups in terms of fasting insulin, HOMA-IR, and MFO after the inter-day CT program. These improvements were comparable in terms of both percentage change and effect size.

Interestingly, the individual response analysis indicated that a higher percentage of participants in the EM-G responded positively in terms of fasting insulin and HOMA-IR compared to the exercise-only group (E-G). Conversely, a higher percentage of MFO responders was observed in the E-G than in the EM-G. The maximal oxygen uptake (VO2_max_) showed significant improvements only in the E-G, with a higher percentage of responders than in the EM-G. Both the percentages of fat mass and body mass were significantly reduced across all groups. Body mass decreased in the E-G, EM-G, and M-G, with the highest percentage of responders in the E-G. The reduction in the percentage of fat mass was significant in the E-G. These findings did not support our primary hypothesis, which posited that CT without metformin would have a more significant impact on metabolic markers and cardiorespiratory fitness than CT combined with metformin.

### 4.1. Insulin Sensitivity (HOMA-IR)

Our findings indicated a significant improvement in the HOMA-IR index in both intervention groups, the E-G and EM-G, compared to the M-G. This trend is supported by Malin et al. [16], who observed the effects of an endurance (60–75 min at 70% of the maximal heart rate) and resistance (60–75 min at 70% of 1RM) training program, with and without metformin, in men and women with IR for 12 weeks. The authors documented that the improvement in insulin sensitivity (by hyperinsulinemic–euglycemic clamp) was 25–30% greater after exercise without metformin, although this difference was not significant.

In contrast, Abdelbasset [46] compared the effects of endurance and resistance exercise programs in combination with metformin in patients with T2DM. A total of 57 patients were included in the study, which was conducted in three groups: an endurance exercise group with metformin (50–70% HRmax, for 40–50 min), a resistance exercise group with metformin (50–60% of 1RM, for 40–50 min), and a metformin-only group. After the 12-week period, the exercise groups exhibited significant improvements in fasting blood glucose, glycosylated hemoglobin (HbA1c), the HOMA-IR, and VO2_max_, with the endurance group demonstrating the most pronounced enhancement. The metformin-only group demonstrated a significant reduction in fasting glycemic levels. In conclusion, the combination of resistance exercise and metformin was more efficacious than endurance exercise alone in the management of T2DM.

Walton et al. [21], in a randomized, double-blind, placebo-controlled trial, investigated the effects of metformin on muscle hypertrophy in response to a progressive resistance program in older adults (n = 47; age range, 65–75 years). The participants were randomly assigned to receive metformin or a placebo for 24 weeks. During this time, all participants underwent progressive resistance training. The measures used were muscle cross-sectional area and strength. The results showed that, compared to the placebo group, the M-G had a smaller increase in muscle cross-sectional area and strength after 24 weeks of training. Additionally, the M-G showed higher levels of myostatin, a protein that inhibits muscle growth, and lower levels of satellite cells, which are essential for muscle repair and growth [21].

Consistent with the above, the results show controversial findings, suggesting an attenuating effect of metformin as a function of exercise type (i.e., endurance, resistance, or concurrent) on insulin sensitivity adaptations. Walton et al. [21] suggested that metformin might attenuate muscle hypertrophy in response to progressive resistance training in older adults. These findings have implications for the use of metformin for the treatment of age-related muscle loss and weakness.

In terms of pharmacodynamics, metformin exerts its molecular action by inhibiting complex I, mitochondrial glycerophosphate dehydrogenase, and ATP synthase [19]. This decreases ATP production and increases AMP concentration, which increases the [AMP]/[ATP] ratio. As a result, AMP-activated protein kinase (AMPK) is activated, and insulin sensitivity is increased [19]. However, it is important to note that this molecular mechanism of metformin may antagonize the improvement in insulin sensitivity and cardiorespiratory fitness induced by aerobic exercise due to mitochondrial dysfunction [23]. Interestingly, to date, only theoretical mechanisms have been proposed for the molecular mechanisms associated with CT with or without metformin [47], which highlights the relevance of studies in this direction.

Furthermore, it is pertinent to point out that the methodology of high-volume, moderate, continuous, and frequent endurance training can negatively affect adaptations such as protein synthesis, muscle hypertrophy, and glucose storage capacity [21] induced by resistance training due to the inhibition of the mammalian target of rapamycin (mTOR) protein by the activation of the AMPK protein [48]. In contrast, short, low-volume workouts, such as HIIT or sprint interval training, are thought to have a minor or even no negative effect on molecular adaptations associated with muscle protein synthesis in response to resistance training in a concurrent program [49]. Therefore, it is critical to investigate the mechanisms through which different training modalities interact, especially in combination with metformin, to optimize IR interventions and maximize the benefits of concurrent training.

### 4.2. Maximal Fat Oxidation and Cardiorespiratory Fitness

The MFO results showed improvements in both the E-G and EM-G, with a greater magnitude of change in the E-G (181.1% and 67.5%, respectively). Consistent with our results, Malin and Braun [26] investigated whether metformin could attenuate the ability to increase fat oxidation and free muscle glycogen levels. They evaluated the effect of a 10-week CT program that included aerobic exercise on a bicycle ergometer (60–75 min) twice a week and resistance training (60–75 min at 70% 1RM on the second day of the week) with and without metformin in sedentary adults with chronic glucose intolerance. They found no group differences in MFO but observed an increase in insulin sensitivity, which was significantly greater in the CT group without metformin after 10 weeks of CT. Furthermore, they found that training-induced improvements in insulin sensitivity, as measured by the hyperinsulinemic–euglycemic clamp method, correlated with increased cardiorespiratory fitness (VO2_max_) (r = 0.70; *p* < 0.05) [27].

In contrast, Malin et al. [49] studied resting, during-, and post-aerobic exercise responses in 15 healthy, recreationally active individuals who were treated with 2000 mg/day of metformin or placebo for 8–10 days using a double-blind crossover design. The results showed that metformin at rest increased fat oxidation. However, metformin reduced fat oxidation after the endurance-type exercise protocol during an exercise session on a bicycle ergometer. The authors concluded that in healthy individuals, metformin had opposite actions on fat oxidation [49].

Regarding cardiorespiratory fitness, our results revealed a significant improvement in VO2_max_ in the E-G (+13.3%), whereas the EM-G and M-G showed no significant changes. The EM-G indicated an improvement of 5.26%, and notably, the M-G indicated a decrease of −30.8%. These findings were described by Cadeddu et al. [22], who observed a decreasing trend in VO2_max_ in the metformin-only treated group. Specifically, 75 patients (35 men and 40 women [46 ± 11 years]) with IR were divided into three groups: metformin and training (30–50 min of aerobic exercise on a bicycle ergometer at 60–80% of the baseline heart rate) with and without metformin. After 12 weeks, a significant increase in cardiorespiratory capacity was observed in the exercise groups with and without metformin (*p* < 0.01), with no significant differences between the groups analyzed. This variability in results can be largely attributed to methodological differences between studies, for example, acute exercise sessions with chronic effect interventions and assessments of healthy individuals versus those with IR. In addition, exercise programs vary between interventions, including endurance exercise or CT.

Obese or overweight patients with IR often have decreased endurance or cardiorespiratory fitness, which could also imply a reduction in fat oxidative capacity [1]. Among the benefits of physical exercise, it has been shown to improve oxygen uptake, transport, and utilization and thus VO2_max_, potentially improving fat oxidative capacity [50]. Metformin is also effective in reducing hepatic glucose production, thereby promoting body fat oxidation [49,51,52,53,54]. In addition, metformin can reduce visceral fat by promoting fat oxidation and thermogenesis [55]. This finding suggests that the interaction between metformin and exercise may intensify fat oxidation during exercise and potentially increase the effect of exercise on IR. However, it is important to keep in mind that metformin acts directly on mitochondria, altering the balance between mitochondrial coupling and uncoupling reactions and, consequently, cellular bioenergetics [56]. This effect could affect both cardiorespiratory capacity and fat oxidation capacity, rendering cells energetically inefficient and raising questions about the role of metformin as an enhancer or inhibitor of exercise adaptations to improve IR in this population [19].

### 4.3. Inter-Individual Variability

In addition to the overall results, our study examined the interindividual responses (Rs) of the examined groups. For the HOMA-IR, no Rs were found in the M-G, whereas 100% of the Rs were recorded in the EM-G (n = 6). The difference in the proportion of the Rs was statistically significant (*p* < 0.001) (see Figure 2). However, when comparing the M-G with E-G, no significant difference was found between 28.5% of the Rs (n = 2). As for fasting blood glucose, 14.2% of the Rs (n = 1) were documented in both the M-G and E-G. In the EM-G, the Rs were 50% (n = 3). No significant differences were found in the proportions of the groups analyzed. These results were similar to those reported by Alvarez et al. [27]. The authors evaluated the effects of high-intensity intervallic training and a resistance program on IR in women (n = 30; range: 20–40 years). The participants were randomly assigned to one of the following three groups: high-intensity interval training, a resistance program, and the control. Changes in insulin sensitivity, glucose uptake, and lipid profiles were measured. After 12 weeks, both the high-intensity interval training group and the resistance program group showed improvements in insulin sensitivity compared to the control group. Specifically, the HIIT group showed a 21% improvement in insulin sensitivity, while the resistance program group showed a 12% improvement. They concluded that both high-intensity interval training and resistance programs are effective in improving insulin sensitivity in women with IR.

In the case of the VO2_max_, no Rs were observed in the M-G. However, 42.8% of the R values (n = 3) were recorded in the E-G and 16.6% in the EM-G (n = 1). No significant differences were found in the proportions of the groups analyzed. These results align with those described by Seward et al. [57], who examined the efficacy of a community-based, personalized exercise program aimed at mitigating the severity of metabolic syndrome and, consequently, reducing T2DM and cardiovascular disease. Seward et al. [57] reported on 150 physically inactive individuals (range: 18–83 years). The participants were randomly divided into a control group (n = 75), who were asked to maintain their usual routine, and a treatment group (n = 75). The latter completed a 12-week personalized fitness training program. After 12 weeks, the authors reported interindividual variability in responses to the exercise program in terms of the VO2_max_. Although some individuals achieved significant improvements, others showed minimal changes or decreases in VO2_max_. Importantly, however, some participants experienced an increase ≥ 30%, underscoring the potential for improvement in aerobic capacity with appropriate intervention. In addition, the authors found that certain demographic and clinical characteristics, such as a younger age, a lower basal VO2_max_, and the presence of certain comorbidities (e.g., hypertension and obesity), were associated with greater improvements in cardiorespiratory capacity. This phenomenon has been extensively analyzed in a review by Williamson et al. [58], who noted that genetic differences, muscle fiber composition, hormonal responses, muscle architecture, neuromuscular coordination, body composition, nutritional status, presence of chronic diseases, and medication use can significantly influence individual variations in VO2_max_ in response to physical training.

Regarding MFO, 42.8% of the Rs (n = 3) were recorded in the E-G, whereas 14.2% of the Rs (n = 1) were recorded in both the EM-G and M-G. No significant differences were observed in the proportions between the groups. In contrast, Malin and Stewart [1] and Bonafiglia et al. [59] in recent reviews highlighted the significant interindividual variability in responses to exercise therapy and metformin use, respectively. This variability may be influenced by factors such as gut microbiota composition, gut epithelial barrier integrity, diet, genetic variants, and miRNA and protein expression [59]. Changes in gut microbiota and differences in drug metabolism may affect the efficacy of metformin. However, responses to physical exercise may vary due to factors such as genomic instability, telomere length, mitochondrial function, and chronic inflammation [59]. In addition, nutritional, stress, sleep quality, and lifestyle factors may contribute to interindividual variability in response to metformin treatment and the efficacy of physical and nutritional training [58,59]. This knowledge underscores the importance of considering personalization in health interventions to optimize their efficacy.

### 4.4. Limitations

It is relevant to note that our study was not without limitations. This study used indirect measurements related to metabolic and body composition markers, whose results should be interpreted with caution. The HOMA-IR index is an indirect measure to determine insulin sensitivity, albeit associated with pancreatic β-cells. Therefore, the interpretation of the results related to muscle metabolic peripheral adaptations should be analyzed with caution. However, its correlation with the gold standard “hyperinsulinemic–euglycemic glucose clamp test”, whose high cost and complexity limit its practical application, makes the HOMA-IR a useful measure in clinical and epidemiological studies.

In addition, it is recognized that the optimization of body composition is determined not only by physical exercise but also by nutrition. Therefore, to interpret the results regarding the decrease in body mass and fat mass percentage after 12 weeks of inter-day CT requires the control of nutritional habits, although our study emphasized the adaptations of metabolic markers and cardiorespiratory fitness. Thus, despite the monitoring of lifestyle changes by phone calls during the 12 weeks, we acknowledge its relevance.

Moreover, bioelectrical impedance is an indirect technology to measure body composition and whose results depend on the state of hydration, which may underestimate or overestimate the results obtained. Although, it is relevant to mention that %FM correlates with its counterpart determined by x-ray absorptiometry.

Finally, we recognize the inherent biological differences between men and women, whose analysis could have affected the results analyzed. However, to remedy this limitation, participant eligibility was rigorous, considering their demographic and disease-related similarities.

### 4.5. Perspectives and Future Studies

The prescription of physical exercise as a medicine is currently recognized to prevent and treat diseases associated with unhealthy lifestyle habits in the population. Therefore, this study contributes to advancing the understanding of inter-day distributed concurrent training with an emphasis on continuous and interval aerobic exercise sessions during pharmacological treatment with metformin. Thus, our findings highlight that a combination of exercise and metformin may offer benefits that would outweigh those of metformin alone in the treatment of IR.

Accordingly, from a research perspective, retrospective design studies offer answers when logistics are limited for pure experimental design studies. Furthermore, the incorporation of analyses based on the principle of individuality of physical exercise enriches the documented results by allowing the determination of individual changes in relation to the study group. In addition, this methodology can serve for clinical analysis as part of the control of the treatment of these diseases. In turn, it is relevant to identify the factors that generate interindividual variability in the responses to exercise and metformin independently, since understanding these elements will allow a more effective personalization of interventions, representing a challenge and an opportunity for future research and clinical practice.

Future studies could focus on analyzing the intra-session effects of CT in combination with metformin to determine whether the distribution of CT sessions, and thus their effects, could have more effective group and inter-individual adaptations. Similarly, this research design could be applied to other pathologies using pharmacotherapy in conjunction with physical exercise.

These results reaffirm the crucial role of incorporating physical exercise, alone or in combination with metformin, in the improvement of IR, compared to the use of metformin as the sole pharmacological treatment. However, more detailed research is required to unravel the complex interactions between the different types of physical exercise, metformin, and IR, which could allow more precise prescription of the most beneficial type of exercise when medication cannot be discontinued.

## 5. Conclusion

The 12-week inter-day concurrent training program, whether combined with metformin or not, was similarly effective in improving metabolic markers in patients with insulin resistance as metformin treatment alone. Both exercise groups demonstrated a significant reduction in insulin sensitivity by the HOMA-IR and an increase in maximal fat oxidation. Meanwhile, exclusive pharmacological treatment with metformin markedly decreased cardiorespiratory fitness, and consequently, fat oxidation. These findings highlight the importance of incorporating inter-day concurrent training as part of metformin treatment. In this sense, in the middle or long term, a gradual reduction or removal of the medication should be considered as long as they maintain a healthy lifestyle, including concurrent training. Additionally, healthcare professionals should carefully consider the specificity of the exercise type and its potential adaptive effects, the distribution of training sessions, frequency, and the inter-individual response within this population.

## Figures and Tables

**Table 1:** Effects of concurrent training on metabolic and cardiorespiratory fitness and body composition outcomes (n = 20).

Outcomes	Group	Pre	Post	∆%	ES (Pre vs. Post)	R_S_ (%)	Group by Time (F_2,17_; *p* Value; η^2^_p_)
Body Mass (kg)	M-G	94.23 ± 13.93	89.39 ± 14.77 *	−5.15	0.30	3 (33.3%)	11.961, 0.13, 0.088
	E-G	85.27 ± 19.67	77.23 ± 19.06 *	−9.43	0.73	5 (71.4%)	
	EM-G	88.13 ± 12.66	80.00 ± 13.44 *	−9.21	0.05	5 (83.3%)	
%Fat Mass	M-G	41.14 ± 12.88	36.26 ± 13.67 *	−11.87	0.45	5 (71.4%)	2.437, 0.11, 0.22
	E-G	35.21 ± 8.200	27.29 ± 6.522 *	−22.52	0.83	7 (100%)	
	EM-G	41.53 ± 10.93	34.62 ± 11.30 *	−16.65	0.63	4 (66.7%)	
Fasting Insulin (mg/dl)	M-G	15.63 ± 3.74	15.73 ± 5.86	+0.63	0.02	0	34.059, 0.001, 0.88 *
	E-G	16.4 ± 3.31	2.92 ± 1.89 *✤	+82.1	4.06	5 (71.4%)	
	EM-G	18.5 ± 3.45	2.76 ± 1.49 *✤	+85	4.54	6 (100%)	
HOMA -IR	M-G	3.24 ±0.94	3.24± 1.28	0	0	0	35.597, 0.001, 0.80 *
	E-G	3.57 ± 0.75	0.62 ± 0.40 *✤	+82.6	−3.91	3 (42.8%)	
	EM-G	4.1 ± 0.93	0.63 ± 0.37 *	+84.6	3.69	6 (100%)	
Fasting Glycemia (mg/dl)	M-G	83.43 ± 8.18	82.86 ± 6.89	−0.68	0.06	2 (28.5%)	1.318, 0.294, 0.134
	E-G	88.2 ± 3.98	88 ± 3.36	−0.22	0.07	3 (50%)	
	EM-G	89.83 ± 6.61	86.25 ± 7.53	−3.98	−0.54	3 (50%)	
MFO (g/h)	M-G	6.07 ± 5.23	6.13 ± 4.72	+0.09	0.01	1 (14.2%)	4.541, 0.026, 0.348 *
	E-G	4.5 ± 5.89	12.65 ± 3.94 *	+181.1	1.38	3 (42.8%)	
	EM-G	6.66 ± 5.93	11.16 ± 4.10	+67.5	0.75	2 (33.3%)	
VO2_max_ (L/min)	M-G	2.98 ± 2.06	2.06 ± 0.66	−30.8	−0.05	0	3.379, 0.058, 0.284
	E-G	2.1 ± 0.93	2.38 ± 1.13 *	+13.3	0.30	3 (42.8%)	
	EM-G	1.71 ± 0.51	1.8 ± 0.55	+5.26	0.16	1 (16.6%)	

M-G, metformin group; E-G, exercise group; EM-G, exercise–metformin group. Means: ✤ (statistically significant (*p* < 0.05) to M-G); *: significant changes in time factor. (*p* < 0.05).

## Data Availability

Data are available upon request from the corresponding authors.
